# The feasibility and acceptability of the provision of alcohol screening and brief advice in pharmacies for women accessing emergency contraception: an evaluation study

**DOI:** 10.1186/1471-2458-14-1139

**Published:** 2014-11-05

**Authors:** Sally Brown, Emily Henderson, Claire Sullivan

**Affiliations:** School of Medicine, Pharmacy and Health, Durham University, Queen’s Campus, Thornaby on Tees, TS17 6BH UK; Public Health, Durham County Council County Hall, Durham, DH1 5UJ UK

**Keywords:** Alcohol brief intervention, Community pharmacy, Women, AUDIT questionnaire, Emergency contraception, UK

## Abstract

**Background:**

It is widely accepted that excessive drinking contributes to both health and social problems. There has been considerable interest in the potential of community pharmacies as a setting for health advice, and evidence suggests that interventions by pharmacists can be effective. Research on interventions relating to alcohol consumption in primary care has focused on general practice, and although some evidence exists about the efficacy of pharmacy interventions, little research to date has taken place in the UK. The aim of this study was to evaluate the acceptability of alcohol screening and brief interventions to women accessing emergency hormonal contraception (EHC) in community pharmacies.

**Methods:**

An initiative whereby women who accessed community pharmacies for EHC would be asked to complete an AUDIT questionnaire following their EHC consultation was introduced by a Primary Care Trust (PCT) in the North-East of England. The evaluation incorporated three strands: interviewing pharmacists (n = 14) about the implementation and acceptability of the initiative; interviewing clients (n = 22) identified as “low risk” to understand their perceptions of the initiative; conducting online follow-up surveys with clients in the “risky” group (n = 53) to evaluate the impact of the initiative on their alcohol consumption and contraceptive behaviour, as well as their perceptions of the service.

**Results:**

Pharmacists’ attitudes towards screening were generally positive, although there were organisational obstacles to providing the service. Some felt uncertain about engaging clients in conversation about a sensitive topic. However, clients themselves did not report feeling embarrassed or upset, and most were happy to talk to the pharmacist and be given advice. Most clients felt that the pharmacist was an appropriate person to carry out alcohol screening and advice.

**Conclusions:**

It is feasible for pharmacists to carry out screening and brief advice, and most customers find it acceptable. However, pharmacist take-up of the service and participation in the study was low. Pharmacists were enthusiastic about providing screening and other health promotion services; targeting different population groups for alcohol screening may be more successful. Delivery of the AUDIT tool by pharmacists may not obtain reliable responses from some specific client groups.

## Background

It is now widely accepted that excessive drinking contributes to both health and social problems [1–3]. There is also evidence to indicate that alcohol consumption adversely affects sexual risk taking; in particular, young people who drink and people who binge drink are more likely to have unprotected sex, to contract Sexually Transmitted Infections (STIs), and for women, to have an unplanned pregnancy [4]. Population-level interventions seeking to influence the price and availability of alcohol are likely to be most effective in reducing these problems [5], and these may be complemented by individual-level interventions, delivered in a range of settings. The Department of Health in the UK has recommended that alcohol interventions based in community pharmacies should be piloted and evaluated [6].

Research on brief alcohol interventions in primary care has tended to focus on general practice, or on particular populations. A systematic review of studies focussing on interventions with hazardous drinkers suggested that Identification and Brief Advice (IBA) has moderate efficacy [7]. Questions remain about its applicability to general populations, and in non-research contexts. IBA was effective in reducing alcohol consumption at 6 and 12 month follow up for people attending primary care facilities but not for alcohol-related problems [8]. Another review identified 29 controlled trials, 24 of which were in general practice and 5 in an emergency setting, and found a clear benefit of IBA for men in reducing alcohol consumption; as the benefit for women was unclear, it suggested that further research is needed which focuses specifically on women [9]. However, a recent study of a large trial in primary care practices suggested that brief intervention provided only limited additional benefit over and above a patient information leaflet [10].

There has been considerable interest in the potential of community pharmacies as a setting for health promotion and advice, partly because of their accessibility and high level of use, and also because of the opportunity presented to provide advice to populations who might not be directly seeking information or advice about specific problems or conditions. There is evidence to suggest that advice and interventions by pharmacists can be effective in promoting health behaviour [11] and in smoking cessation and lipid management for populations at risk of coronary heart disease [12], and that clients find it acceptable and beneficial [11, 13–15]. In addition, pharmacists in New Zealand were found to be keen to take on the role of screening and delivering brief interventions for alcohol [16], while pilot studies conducted in London and Glasgow found that pharmacists were interested in undertaking training in IBA in order to identify and help modify risky drinking behaviour of pharmacy service users [17, 18]; the Glasgow pharmacists also reported positively about the effect of the training on their knowledge of IBA [19].

There is a growing body of research on the effect of IBA in pharmacies. An early literature review [20] identified only three studies carried out in community pharmacies [21–23], two of which showed non-significant reductions in alcohol consumption by clients following brief interventions by pharmacists [21, 23]. A further study investigated whether pharmacists could opportunistically identify patients who were hazardous drinkers [22]. Pharmacists reported that workload, time constraints and embarrassment were potential barriers [21, 23], and that some pharmacists may have been less likely to approach potentially problematic clients [22]. The review concluded that community pharmacies should be included in strategies to reduce alcohol consumption, and that studies are needed to evaluate the effects and cost-effectiveness of community pharmacy-based interventions, and to explore the acceptability of the service to users. A more recent UK study showed that most pharmacy users were positive about an IBA delivered by community pharmacists [24].

These studies suggest that a community pharmacy-based alcohol screening service, incorporating a brief intervention, is broadly acceptable to clients, and has the potential to identify risky drinkers and reduce such behaviour. These studies indicate that community pharmacists are willing to deliver IBA as part of their developing public health role and have the potential to deliver this to client groups across the general population of pharmacy service users.

The Alcohol Use and Disorders Identification Test (AUDIT) is an IBA tool developed by the World Health Organisation [25] for use in primary care settings. It has been widely validated for use in a range of settings, and in particular is recommended for use in community settings [26]. In addition, it has been shown to have good sensitivity and specificity to detect harmful and hazardous drinking amongst people not seeking treatment for alcohol problems. It is sensitive to change, at least in older male patients [27] and is therefore suitable for use in follow-up, allowing changes in drinking behaviour to be measured.

Internet-based follow-up may result in lower consumption and frequency of drinking after six weeks compared to leaflets [28] but studies differ in whether the reduction in hazardous drinking can be maintained [28, 29]. Online questionnaires have been used widely in delivering interventions [28–31] and are seen to be effective [32], but they have not been used in following up the effectiveness of a face-to-face intervention. Data on follow up beyond one year is very limited, but it appears that where there may be a continuing small effect at 4 years [33] this has disappeared at ten years [34].

Evidence seems to indicate, therefore, that IBA works in primary care and that community pharmacies have a great deal of potential; however, the key to successful interventions is yet to be identified, along with whether it works for all groups of heavy drinkers [35]. In addition, it is vital to carry out research that takes into account practitioner views in order for initiatives to reach the client groups they are intended to benefit and to embed brief interventions in practice [36].

In 2009 local Alcohol Harm Reduction Strategies were launched by a Primary Care Trust (PCT) in the study area which aimed to tackle alcohol related harm across the county. As part of the strategy, a pilot initiative was introduced for the use of an AUDIT questionnaire and delivery of IBA in community pharmacies to women presenting for emergency contraception. This was considered as a potential ‘teachable moment’ for a specific client group. The aim of the study was to evaluate the impact of the AUDIT tool on alcohol use and the feasibility of the provision of the advice. This paper discusses the results of the evaluation of the initiative. Both the initiative and the study were developed and managed with involvement of local pharmacists, mainly via the Local Pharmaceutical Committee, giving insights not only into the use of IBA with a hitherto less well studied group, i.e. women [9], but also into the translational aspects in carrying out research with community practitioners.

## Methods

The aim of this study was to evaluate the acceptability of alcohol screening and brief interventions to women accessing emergency hormonal oral contraception in community pharmacies.

The research questions were:

-  Is it feasible and practical for pharmacists to deliver screening and brief advice in the course of a consultation for emergency hormonal contraception?
-  Is the delivery of screening and advice acceptable to pharmacists?
-  Is the delivery of screening and advice acceptable to clients?

### Study design

In order to answer the research questions related to provider and client views of the service, interviews were carried out with 14 pharmacists and22 low-risk drinkers, and an acceptability survey was adminstered to the low-risk drinkers and to 53 risky drinkers. All pharmacists from participating pharmacies were invited to take part. Clients were invited to participate by pharmacists upon completion of the AUDIT questionnaire, and, if interested, asked to provide contact details for the research team.

### Pharmacist interviews

Face-to-face in depth focused interviews were carried out with pharmacists to investigate their views about the delivery of screening and advice, and to ascertain their views on the usefulness of the “teachable moment”. Purposive sampling ensured that a range of respondents was included; in particular those who had taken up the training but not provided the service were interviewed to explore some of the potential barriers to providing the service. In total 14 interviews were completed. They were recorded and fully transcribed. A thematic analysis of the data was undertaken using the framework approach [37]. This is an approach to analysis developed for applied policy research, which allows the exploration of issues of interest as well as allowing for new issues to emerge. All transcripts were read and re-read to identify themes; a framework then was constructed, which was systematically applied to all transcripts.

### Client interviews and survey

Using the AUDIT tool, clients were categorised as “low-risk” drinkers if they scored 7 or less, “risky” if they scored 8–19 and “possible dependence” if their score was 20 or higher. Those in the “possible dependence” group were not given brief advice but referred by the pharmacist on to appropriate services, and were not invited to participate in the research. Clients were asked to provide their age in years and partial residential postcode (to the sector level, e.g. AB12 3xx) to calculate index of deprivation [38], and indicator of socio-economic status. An acceptability survey containing eight questions (see Table 1) was adapted for a pharmacy setting from one which had been used in a survey of attitudes to alcohol screening by dentists, [39] whereby respondents select ‘strongly agree’, ‘agree’, ‘disagree’ and ‘strongly disagree’. The survey was administered to the low-risk group over the phone, one to two weeks after the initial consultation with the pharmacist. Participants in the risky group took the survey approximately three months after the initial consultation with the pharmacist, via a secure Bristol Online Survey, inconjunction with an additional online survey that the research team piloted to assess the effectiveness of the AUDIT tool over a 6-month period (data not reported here). Survey data were imported into SPSS 20. Categories were amalgamated to increase sample size, such that ‘strongly agree’ and ‘agree’ become ‘agree’, and ‘strongly disagree’ and ‘disagree’ become ‘disagree’. One-sample binomial exact tests were conducted using 0.5 as the comparator.

**Table 1:** **Survey of the acceptability of the intervention to clients**

	n (%)	
	Agree/Strongly agree	Disagree/Strongly disagree
1. I was annoyed when the pharmacist asked me about my drinking**	5 (6.7)	70 (93.3)
2. I was upset when the pharmacist asked me about my drinking**	5 (6.7)	70 (93.3)
3. I did not mind being asked about my drinking**	67 (89.3)	8 (10.7)
4. It was embarrassing to be asked about alcohol by the pharmacist**	9 (12.0)	66 (88.0)
5. I was glad that the pharmacist gave me advice about alcohol*	49 (65.3)	23 (30.7)
6. I was happy to have someone to talk to about my drinking	34 (45.3)	38 (50.7)
7. I don’t need any advice about the amount I am drinking**	61 (81.3)	14 (18.7)
I thought that the information the pharmacist gave me was useful**	63 (84.0)	10 (13.3)

*p < 0.01.

**p < 0.001.

### Ethical issues

Informed consent was obtained from all participants, verbally for the telephone interviews and electronically for the survey participants. Data were treated according to the Data Protection Act 1998. Ethical approval for the study was obtained from the School of Medicine and Health Research Ethics Committee, and the County Durham and North Tees NRES Committee North East.

## Results

The findings of the interviews with the pharmacists are considered first; we then discuss the results of the telephone interviews and the survey that consider client attitudes to be asked about alcohol consumption by a pharmacist.

### Pharmacists’ views of the intervention

The discussion which follows is based upon the five key themes emerging from the analysis:

-  Barriers to implementing the intervention
-  Introducing the intervention
-  Engaging clients
-  Impact of the intervention
-  Developing the role of community pharmacy

#### Barriers to implementing the intervention

A number of obstacles were identified which the pharmacists felt hampered their ability to implement the intervention. The two key obstacles were time and paperwork. In some cases, although the number of forms and leaflets had seemed overwhelming at first, it was a barrier that had been overcome:

*I just felt there was just so much paperwork and i was like “Woah!”, I was just overcome with all the paperwork and as I said it made me quite negative about doing it but then I realised, no it should be something good, and then once I got over the paperwork hurdle I was ready to go for it (City centre, multiple, female pharmacist)*

In other cases, pharmacists saw it as yet another form to fill in, and if they were less enthusiastic to begin with, were less likely to persist as the pharmacist above had done.

Time could be an obstacle, firstly because of the competing pressures of working in a busy pharmacy, which applied to some of the smaller pharmacies staffed by a sole pharmacist, as well as the busier city pharmacies. Secondly, where there were many competing demands and the alcohol IBA was seen as yet another one:

*It’s just another burden to be quite honest with you, on top of everything else, you know? We’re that pushed for time as it is (Former mining village, small chain, male pharmacist)*

Although there was a financial payment for taking up the initiative and offering the service, as well as recruiting participants into the study, views on whether this was a sufficient incentive varied. Some pharmacists felt that it was necessary to have some form of financial recompense, but others did not feel the payment was necessary as an incentive:

*It wouldn’t make any difference to me how much we got paid. I would do the service if I felt it was the right thing to do. (Former mining village, small chain, male pharmacist)*

It seems that those pharmacists who are motivated to implement the intervention, a financial incentive is welcome but not vital, and for those who perceive there to be too many barriers, a financial incentive is insufficient to overcome them.

#### Introducing the intervention

Issues around how to introduce the topic and how to manage the process of asking clients about their alcohol consumption were key practical aspects of undertaking the intervention:

*I always say “was alcohol involved?” after that. That’s where that’s my trigger for the form, but they say no, and then I’ll say “do you want to fill out the form?”, and they say “no”, that’s it. (Rural town, single, female pharmacist)*

An alternative opening was to say that everyone was being asked, or the University was doing a study, and this had more positive results:

*I say the University has asked us to help them, and that really helps when you mention the University. (Suburb, small chain, female pharmacist)*

At the training sessions, pharmacists were supplied with a range of supporting material to take away including posters, leaflets and tools to demonstrate alcohol measurement units such as “drink wheels”. The posters, which had been designed for the project by the PCT’s Social Marketing team and the study steering group proved to be a useful “way in” to the potential tricky conversation:

*I’ve had two or three incidents where the poster’s actually led the person to say “oh yeah that’s me”. (Small town, multiple, male pharmacist)*

However some of the bigger chain stores would not allow the pharmacists to display the poster in a prominent position.

One of the main challenges identified by pharmacists, which related to feeling awkward or uncomfortable in introducing the topic, concerned the lack of opportunity to use the tools due to low uptake of services, either because of low demand for EHC or because of high rates of refusal to complete the AUDIT questionnaire. There was a clear difference between those pharmacists who felt they were missing the opportunity to use the tools, and therefore losing the skills they had gained, and those who did have a sufficient client base to become accustomed to using the tools:

*The more you don’t do it, the more and more you kind of, the knowledge kind of just slips away a little bit. (Small town, multiple, female pharmacist)*

#### Engaging clients

The biggest concern expressed by pharmacists both in the development period for the initiative, and during interviews, centred upon the challenges of engaging the clients in what they felt could be a difficult and potentially embarrassing topic. Nevertheless, this was that issue that was most frequently mentioned, and discussed at greatest length, during the interviews with pharmacists.

Many pharmacists said that their clients gave the impression of wanting the EHC consultation to be dealt with swiftly, and not to have to spend any longer in the pharmacy than necessary. In some of the smaller communities, it was suggested that existing relationships where the pharmacist and client knew or recognised each other added to potential embarrassment and reluctance to discuss sensitive issues. Pharmacists were also wary of damaging a potentially fragile relationship of trust:

*I didn’t want to push it too much in case they didn’t want to come back again, in case it ever happened again. ‘Cos in case they get hounded ‘Oh I’m not going to go there ‘cos I’m just going to get hounded and lectured about alcohol’. (Former mining village, multiple, male pharmacist)*

It was felt to be more difficult to broach the subject with older women in particular, and in some cases the pharmacists made a judgement about whether or not to approach the topic with them, based on their knowledge about whether they had a regular partner and whether they were a potential candidate for an alcohol IBA:

*Subconsciously you sort of screen them whether or not it would have been suitable to do it. (Small town, multiple, male pharmacist)*

Younger teenagers were sometimes implicitly excluded from the intervention in a similar way, because the pharmacist deemed them to be “too frightened”, or because they were visibly upset. In other cases it was simply impractical, because the client brought a friend, partner or child with her.

#### Impact of the intervention

The pharmacists who had managed to become engaged with the intervention, mainly as a result of having sufficient clients willing to complete the AUDIT questionnaire, felt that they were having a useful impact on their clients, particularly because many of them were not aware of the amount they were drinking and how that translated into units:

*They all take the advice on board seriously, you know, and you get the impression from their facial expression and the body language that they are concerned and they realise and they will try and do something about it. (City, multiple, female pharmacist)*

The tools provided at the training, particularly the “drink wheel”, were useful in this situation, and although the paperwork involved in the intervention and the study had been seen as overwhelming, pharmacists felt it was helpful to have information materials to give to the client.

#### Developing the role of community pharmacy

Many pharmacists wanted to have the opportunity to offer enhanced services, and to develop their role in the community in terms of offering more health promotion services. Some pharmacists felt that this was increasingly being asked of them and most were keen to offer more services, feeling that it was a satisfying and enjoyable aspect of their role:

*We do enjoy doing all the service and different promotional activity that we do here (Small town, multiple, male pharmacist)*

*It’s job satisfaction isn’t it, it’s fulfilling when you feel you can help somebody more, sort of by discussing things. And if people start realising they can come in more, and it’s more healthcare and preventative rather than just handing out tablets, so I think it’s more health promotion. (Small village, small pharmacy, female pharmacist)*

However, a small minority were reluctant to offer more services mainly because they already felt under pressure, and as discussed above, felt that they were already short of time and overwhelmed by paperwork.

Several of the pharmacists felt that although the initiative itself was a good idea, in that it focussed people’s attention on their alcohol consumption, selecting women who were accessing pharmacies for EHC was not the ideal target population. This was partly their perception that it was a sensitive topic for that population given their experience of the number of women who did not want to complete the AUDIT questionnaire, although as the client data indicates, many of those who did participate did not feel embarrassed or upset. It was suggested that older people (50–70), people on statins or warfarin, and people attending for medicines use reviews were potentially good target populations:

*I think that the target group, maybe it’s not really right because I think there are lots of customers, I can tell them that when I’m doing their medicines use review they tell me they are drinking, I always give them advice. I tell them, you know, what are the consequences of drinking every day. (Suburb, small chain, female pharmacist)*

Since the commissioning of the study reported here, the initiative has developed and pharmacists are now offering AUDIT screening to a wider range of clients including those who present frequently with symptoms or conditions which may be associated with alcohol misuse e.g. gastric problems, falls, high blood pressure, depression/anxiety/stress or during other consultations for medicine use reviews or smoking cessation clinics.

### Client responses

Pharmacists filled out a total of 613 AUDIT forms with clients, of which 13 were incomplete, 9 were classified as ‘possible dependence’ and 339 declined to take part in the research. The final sample was 252, giving a response rate of 41.1%. There were 86 clients classified as ‘low risk’ and allocated to the phone interview, and there were 166 classified as ‘increasing risk’ and allocated to the online survey. Of those who agreed to take part in a telephone interview, 22 (25.6%) were successfully contacted and interviewed. Fifty-three (31.9%) ‘high risk’ drinkers completed the online survey. The median age of the client group, including both ‘low risk’ and ‘increasing risk’ drinkers, was 20.0 years (19, 21) and the range was 15 to 48 years old (n = 75). Seventy-one per cent of participating clients reported as living in areas classified in the decile of highest deprivation (n = 71).

### Clients’ views on the intervention

The results from the acceptability survey are presented in Table 1. Responses were approximately equally distributed over the ‘agrees’ and ‘disagrees’ only for statement 6.

The majority of clients were not annoyed, upset or embarrassed when the pharmacist asked about their drinking. Likewise, most clients did not mind being asked about their drinking and were glad to receive advice. Most clients thought the advice given by the pharmacist was useful, but also felt they did not need any advice about drinking. Most clients felt that the pharmacist was an appropriate person to carry out screening and advice.

## Discussion

Pharmacists’ attitudes towards screening and giving brief advice were generally positive, which is consistent with findings of other studies both in the UK and New Zealand [16–19]. Organisational obstacles to providing the service, such as lack of time, unfamiliarity with the tool, and pressure of competing demands in a busy pharmacy, meant that some had not had enough practice to feel comfortable with the tools, and this was a major influence on whether or not they became discouraged. Some felt uncertain about engaging clients in conversation about a sensitive topic. During the development of the intervention and the study, a great deal of time was spent discussing how the topic could be introduced, as it was felt to be potentially embarrassing for both pharmacist and client. Various methods of beginning the conversation were suggested, such as using a poster as a prompt, or using one of a number of opening remarks. One of the possible opening lines discussed at the training sessions was “was alcohol involved in the reason for you needing EHC?” and although it was strongly suggested that use of this question would potentially stigmatise and embarrass clients and result in a very low response rate, some pharmacists persisted in using this as an opening line, and these pharmacists had low response rates. In fact, as the data from the client interviews and survey shows, the clients are not usually embarrassed or upset, and generally did not mind being asked about alcohol. In addition, rather than screening all women requesting EHC, some pharmacists chose not to screen some women because they were perceived as “not the type” to drink too much, and some approached the consultation by asking a question which was more likely to result in a negative answer, i.e. did alcohol lead to the need for EHC?, which fed into a very low uptake for those pharmacists. At a time when pharmacists are being encouraged by the Department of Health to offer more health promotion services to their clients, the implication is that some will find it difficult or unattractive to develop these services, although others are very enthusiastic and see it as a key part of their role. The fees offered were not considered to be a major incentive; although they may have been set too low to be effective, it was felt by some pharmacists that some form of incentive was necessary as they were being asked to take on more work. Rather than a purely financial motivation, participating pharmacists were motivated by feeling that they were making a difference and enjoying the opportunity to help people. Again, this is consistent with studies that show that pharmacists can have an impact with a range of health promotion initiatives [11, 12].

Uptake by pharmacists of the opportunity to offer the screening service was low, despite the number of pharmacists trained, and the enthusiasm and encouragement of the Local Pharmaceutical Committee and the Steering Group. Clients’ attitudes to being offered screening were also largely positive, and they felt that it was appropriate to be asked about alcohol consumption by pharmacists. Again, this is consistent with a previous study [24]. As evidence for the impact of applying the AUDIT tool to women is limited [9], our study adds to the limited body of literature on the topic.

### Strengths and limitations of the study

The study has usefully highlighted to pharmacists that the potentially sensitive issue of discussing alcohol use while attending the pharmacist for EOC is something that women would nonetheless feel comfortable discussing. It also showed that it is possible to recruit women seeking EOC to take part in a study of IBA for alcohol use, and offering financial incentives to participants is helpful in increasing participation. However, fewer were recruited than was originally anticipated, and this was due to smaller numbers of pharmacists taking up the initiative than had originally been expected. The response rate to the client survey was low, and thus may have introduced response bias to the sample. However the study results demonstrate the acceptability to clients of alcohol screening and brief advice within a pharmacy setting.

## Conclusions

This study shows that it is feasible for pharmacists to carry out screening and brief advice, and that clients find it acceptable. However, pharmacist take-up of the service and participation in the study was low, which may be a result of targeting the service to this particular client group which led to some pharmacists not feeling that they were getting experience in the use of the tool. Those pharmacists who were using the tool more frequently tended to feel more confident in the delivery of the intervention. Pharmacists were enthusiastic about providing alcohol screening and other health promotion services. Targeting different population groups (e.g. people prescribed Warfarin, patients needing medicines use reviews) may be more successful in terms of integrating the use of the AUDIT tool into practice as well as increasing the competence of the pharmacists in its use.

The study also showed that clients find it acceptable to be asked about alcohol consumption by pharmacists, and do not, on the whole, find it embarrassing. This is an important finding as one of the key barriers to delivery of the intervention was the perception by some pharmacists that the clients would find this a sensitive topic; that clients do not find it a difficult topic should be emphasised as part of ongoing training and introduction of IBA initiatives.

The design of future studies of IBA for alcohol use delivered by pharmacists should consider the method of delivery of IBA on pharmacy premises, and the timing and availability of training for pharmacists in delivering IBA. Further research could also be carried out on the use of the AUDIT tool and IBA in primary care settings beyond general practice and pharmacies.

## Abbreviations

EHC: Emergency hormonal contraception
IBA: Intervention and brief advice
PCT: Primary care trust
STI: Sexually transmitted infection.
